# Isolation of *Schineria* sp. from a Man 

**DOI:** 10.3201/eid1304.061255

**Published:** 2007-04

**Authors:** Laurent Roudiere, Hélène Jean-Pierre, Christine Comte, Isabelle Zorgniotti, Hélène Marchandin, Estelle Jumas-Bilak

**Affiliations:** * University Montpellier 1, Montpellier, France; † Centre Hospitalier Universitaire de Montpellier, Montpellier, France

**Keywords:** Myiasis, Schineria sp., bacteremia, maggot, 16S ribosomal RNA gene, letter

**To the Editor:** *Schineria larvae* has been isolated from maggots of the fly *Wohlfahrtia magnifica* (*1*), which cause myiasis in animals and people in Eurasia and northern Africa. In industrialized nontropical countries, a range of species in the order Diptera cause facultative myiasis in patients with neglected wounds (*2*). Since the recent description of *S. larvae*, *Schineria* sp. isolates and clones have been detected in diverse environmental and animal sources, but in all cases a relation with flies could be established. We describe a case of bacteremia due to *Schineria* sp. in a human patient with myiasis.

In July 2005, a 39-year-old homeless man with medical history of polyneuropathy related to alcohol abuse was examined at Montpellier Hospital, Montpellier, France, and found to be in poor general health and to have an abnormal electrocardiogram, mild fever (38°C), metabolic disorders, increased C-reactive protein (254 mg/L) and fibrinogen (18.23 µmol/L), and a normal leukocyte count (7.8 × 10^9^/L). Removal of his shoes and socks, which he had worn continuously for 2 months, showed advanced maceration of his feet (trench foot) with wounds invaded by maggots. The following organisms were found in wound samples: *Proteus mirabilis*, *Providentia stuartii*, group G *Streptococcus*, *Streptococcus* sp., and *Enterococcus* sp. Aerobic blood culture, after 2 days of incubation, was positive for a gram-negative rod, strain ADV1107.05. Subculture on MacConkey medium showed positive reactions for oxydase, catalase, and gamma-glutamyltransferase. Positive malate reaction with API 20NE system (bioMérieux, Marcy l’Etoile, France) identified the strain as *Oligella urethralis,* whereas VITEK2 (bioMérieux) with ID-GN card failed to identify the strain. Disk diffusion assay showed the strain to be susceptible to β-lactams, aminoglycosides, fluoroquinolones, tetracyclines, erythromycin, rifampin, and colistin but resistant to nalidixic acid and fosfomycin. Local therapy of debridement, bandaging, and sulfadiazin argentic, along with systemic antimicrobial therapy (ofloxacin 400 mg/day plus cefotaxime 6g/day) for 2 weeks, led to clinical improvement and sterilization of the blood cultures. The local therapy was continued, and ofloxacin (400 mg/day) was prescribed for 15 days while the patient was in a rehabilitation center. 

In October 2005, the patient was readmitted with the same symptoms. *P. mirabilis*, group A and group G streptococci, *Morganella* sp., *Bacteroides fragilis*, and *Candida albicans* were cultured from maggot-invaded wounds. Aerobic blood culture, after 1 day of incubation, was positive for strain ADV4155.05, which displayed the same phenotype as strain ADV1107.05 except for tetracycline resistance. Clinical improvement was observed after 2 weeks of the same local and systemic treatments as initially prescribed. The patient was transferred to an addiction care center and received oral antimicrobial therapy (ciprofloxacin 500 mg/day plus amoxicillin/clavulanic acid 3 g/day) for 20 days.

The 16S rDNA amplification and sequencing were performed with universal primers 27f and 1492r as described (*3*). The 1,414-bp sequences of the 2 isolates were identical and showed similarity level of 99.6% with the sequence of *Schineria* sp. 010793816 isolated from human urine (M. Vaneechoutte, pers. comm.) but only 98.3% with *S. larvae* L1/68^T^ 16S rDNA. This finding differed from the biochemical identification and underlined the usefulness of sequencing to precisely identify gram-negative bacilli that assimilate only a few sugars. Phylogenetic analysis linked the 2 strains to the genus *Schineria* in the class Gamma Proteobacteria (Figure). However, whether the isolates are species *S. larvae* remains in doubt. Enterobacterial repetitive intergenic consensus–PCR and repetitive extragenic palindromic–PCR fingerprints (*6*) showed that the 2 strains were unrelated, thereby demonstrating that the second episode of bacteremia was a reinfection with a new strain and not a relapse.

**Figure:**
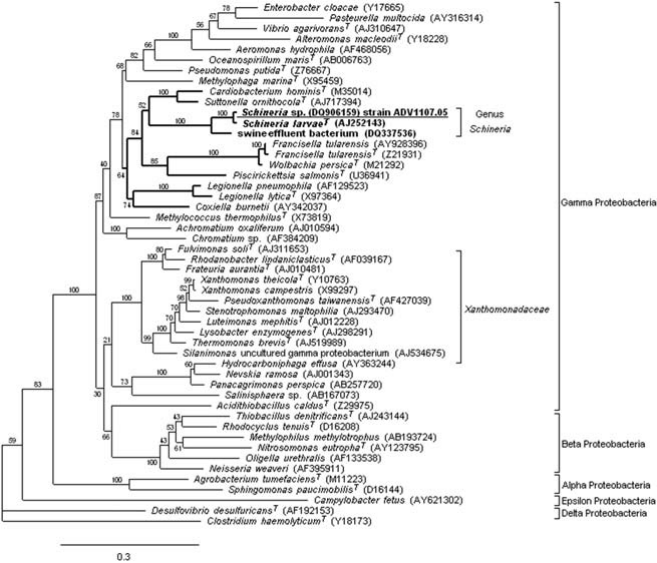
Maximum likelihood (ML) 16S rRNA gene phylogenetic tree showing the placement of the genus *Schineria* (**boldface**) and the isolate ADV1107.05 (underlined) in the phylum Proteobacteria. To reconstruct this tree, we used the strain ADV1107.05 sequence (DQ906159, 1441 bp) and 49 sequences selected from the GenBank database: 38 among the 15 orders of the Gamma Proteobacteria, 6 for the Beta Proteobacteria, 2 for the Alpha Proteobacteria, 1 for Delta Proteobacteria, 1 for Epsilon Proteobacteria and *Clostridium haemolyticum* (used as the outgroup organism). Accession nos. are in brackets. Alignment was performed with ClustalW 1.83 (*4*). ML phylogenetic analysis was performed by using PHYML v2.4.4 (*5*) with the general time-reversible plus gamma distribution plus invariable site (GTR + Γ + I) model found to be most appropriate according to Akaike information criteria. Bootstrap values given at the nodes are estimated with 100 replicates. The scale bar indicates 0.3 substitutions per nucleotide position. Strain ADV4155.05 sequence (DQ906158, 1414 bp) is not reported because it was identical to ADV1107.05. Trees were also obtained by distance methods (JC69, F84, and GTR models, and neighbor-joining), by parsimony, and by Bayesian inference. In all instances the genus *Schineria* branched out of the *Xanthomonadaceae* cluster.

The 16S rDNA of our isolates is most related to an uncultured bacterium found in swine waste (*7*), but its presence in such an environment could be correlated with fly larvae proliferation. Because of the lifestyle of *Schineria* sp., thinking that the strains in our patient originated from his wounds’ maggots is reasonable. Unfortunately, the maggots were thrown away and could be neither analyzed nor identified. *Schineria* sp. could not be cultivated from the patient’s wounds, perhaps because of its close association to larvae or to the abundant associated flora. Despite the presence of virulent bacteria in the wounds, *Schineria* sp. was the sole bacterium recovered from blood during the 2 independent episodes of bacteremia, which suggests its invasive potential. Invasiveness may be enhanced by the maggots’ acting as a vector as they move through the necrotic tissues toward the bloodstream. Invasiveness also may be a specific characteristic of the bacterium; phylogenetic methods placed the genus *Schineria* in a subgroup that included human pathogens *Cardiobacterium*, *Francisella*, *Coxiella*, and *Legionella*. Indeed, all the phylogenetic methods tested excluded *Schineria* spp. of the family *Xanthomonadaceae* (Figure), which conflicts with current classification (*8*).

No report has described bacteremia following myiasis with facultative parasites, but investigations of bacteria in reported myiasis cases have been conducted on cutaneous lesions and never on blood (*9*). Because of this association between maggots and risk for bacteremia, blood cultures should be performed for patients with myiasis and poor hygiene. Moreover, germ-free maggots bred for biosurgery use (*10*) should be checked, by molecular methods, for the absence of *Schineria* sp.
